# Sleeve Gastrectomy and Gastric Bypass Impact in Patient’s Metabolic, Gut Microbiome, and Immuno-inflammatory Profiles—A Comparative Study

**DOI:** 10.1007/s11695-025-07708-9

**Published:** 2025-01-28

**Authors:** Andre Lazaro, Igor Tiago, Joao Mendes, Joana Ribeiro, Antonio Bernardes, Fernando Oliveira, Fernando Regateiro, Francisco Caramelo, Henriqueta Silva

**Affiliations:** 1 General Surgery Unit, Centro Hospitalar E Universitário de Coimbra, ULS Coimbra, Coimbra, Portugal; 2 Coimbra Institute for Clinical and Biomedical Research (iCBR) Area of Environment, Genetics and Oncobiology (CIMAGO), Faculty of Medicine, University of Coimbra, Coimbra, Portugal; 3 Department of Life Sciences, Centre for Functional Ecology (CFE)—Science for People & the Planet, , University of Coimbra, Coimbra, Portugal; 4 Laboratory of Sequencing and Functional Genomics of UC Genomics, Faculty of Medicine, University of Coimbra, Coimbra, Portugal; 5 Faculty of Medicine, University of Coimbra, Coimbra, Portugal; 6 Laboratory of Biostatistics and Medical Informatics (LBIM), Faculty of Medicine, University of Coimbra, Coimbra, Portugal

**Keywords:** Sleeve gastrectomy, Gastric bypass, Impact, Metabolic, Gut microbiome, Immuno-inflammatory profiles

## Abstract

**Background:**

Bariatric surgery is the most long-term effective treatment option for severe obesity. The role of gut microbiome (GM) in either the development of obesity or in response to obesity management strategies has been a matter of debate. This study aims to compare the impact of two of the most popular procedures, sleeve gastrectomy (SG) and Roux-en-Y gastric bypass (GB), on metabolic syndrome parameters and gut bacterial microbiome and in systemic immuno-inflammatory response.

**Methods:**

A prospective observational study enrolled 24 patients with severe obesity, 14 underwent SG and 10 GB. Evaluations before (0 M) and 6 months (6 M) after surgical procedures included clinical and biochemical parameters, expression of 17 immuno-inflammatory genes in peripheral blood leukocytes, and assessment of gut microbiome profile using 16 s rRNA next-generation sequencing approach. Statistical significance was set to a *p* value < 0.05 with an FDR < 0.1.

**Results:**

A significant and similar decrease in weight-associated parameters and for most metabolic markers was achieved with both surgeries. Considering the gut microbiome in the whole study population, there was an increase in alpha diversity at family-level taxa. Beta diversity between SG and GB at 6 M showed near significant differences (*p* = 0.042) at genus levels. Analysis of the relative abundance of individual taxonomic groups highlighted differences between pre- and post-surgical treatment and between both approaches, namely, a higher representation of family *Enterobacteriaceae* and genera *Veillonella* and *Enterobacteriaceae_unclassified* after GB. Increased expression of immune-inflammatory genes was observed mainly for SG patients.

**Conclusions:**

We conclude that SG and GB have similar clinical and metabolic outcomes but different impacts in the gut bacterial microbiome. Results also suggest reactivation of immune response after bariatric surgery.

**Supplementary Information:**

The online version contains supplementary material available at 10.1007/s11695-025-07708-9.

## Introduction

The obesity pandemic has become a concern for healthcare providers and health policies worldwide due to its dramatic increase in the last few decades (WHO 2022) [1]. Metabolic syndrome is the hallmark of pathological obesity. It may be described as a metabolic and immuno-inflammatory disorder involving different biological organs and systems such as the gut, liver, adipose tissue, and brain; cardiovascular, endocrine, and immune systems; and patient microbiome [2]. The role of gut microbiome (GM) in either the development of obesity or in response to obesity management strategies has been a matter of debate. Animal model experiments showed that gut microbiota transplantation influences weight and metabolism [3]. Studies in patients with obesity describe a state of gut dysbiosis characterized by reduced microbial diversity and microbial gene richness, an increase in the relative abundance of the phylum *Firmicutes* (renamed to *Bacilliota*) vs *Bacteroidetes* and *Proteobacteria* phylum, and variations in the abundance of specific bacteria [4–8]. Gene functional analysis of the microbiota of normal-weight individuals shows a lower potential to extract energy from the diet and an increased ability to produce higher levels of short-chain fatty acids like propionate, butyrate, or acetate and also of hydrogen and methane [7].

Metabolic or bariatric surgery is the most long-term effective treatment option for severe obesity [9]. Sleeve gastrectomy (SG) and *Roux-en-Y* gastric bypass (GB) are two of the most performed operations. Bariatric surgery improves weight loss and the reversion of metabolic syndrome through several mechanisms beyond the simple restriction of food intake and nutrient malabsorption [9, 10]. For instance, the increase of glucagon-like peptide 1 (GLP-1) and gastric inhibitory polypeptide (GIP) release by gut epithelium associated with *Roux-en-Y* gastric bypass (GB) and other procedures that exclude the duodenum from the passage of food is of utmost importance to improve glucose homeostasis and reduce insulin resistance 11. Gut microbiota studies after bariatric surgery reveal favorable profiles characterized by partial reversion of obesity pattern, though with variability in the changes of specific taxonomic groups described by different authors [5, 12–15]. Remodeling of the microbial community was described within the first 3 months after surgery and persists after 9 years [15–18]. The post-bariatric surgery GM promotes a reduced availability of energy for mucosal uptake and associates with the improvement of obesity-related low-grade inflammation [19–21]. Enrichment in *Akkermansia,* a mucin-degrader from *Verrucomicrobia* phylum, and in bacteria from the *Firmicutes* phylum known to form biofilms and to contribute to lactate metabolism, is believed to improve colon epithelial barrier and integrity [22]. Moreover, germ-free mice transplanted with feces from bariatric surgery patients gained less fat when compared to reciprocal mice receiving feces from patients with obesity [18]. Notwithstanding, controversy remains, and some studies suggest that bariatric surgery–associated GM changes may be independent of weight loss or caloric restriction [18]

This study aims to compare SG and GB impact on metabolic syndrome regression, gut bacterial microbiota, and systemic immuno-inflammatory response.

## Material and Methods

### Patients

A prospective observational study was conducted in a high-volume obesity surgery unit. Patients were consecutively enrolled and selected for GB and SG in alternate order. All patients were evaluated 1 week before surgery (0 M) and 6 months after surgery (6 M).

The study was performed according to the ethical principles governing medical research and human subjects as laid down in the Helsinki Declaration (2002 version, www.wma.net/e/policy/b3.htm), with the approval of the Institutional Review Board/IRB (Ethics Committee) (approval number 143–17; date of approval 01/10/2018). All patients were informed of all procedures and signed a written informed consent.

Inclusion criteria were body mass index (BMI) ≥ 40 kg/m^2^ and no obesity-related complications or BMI between 35 and 40 kg/m^2^ with obesity complications such as type 2 diabetes mellitus (DMT2), arterial hypertension, dyslipidemia, obstructive sleep apnea, or osteoarticular degenerative disease.

Exclusion criteria included the following: incomplete clinical or laboratory data, chronic inflammatory diseases imposing anti-inflammatory medication, occurrence of infectious or inflammatory diseases during the study, intake of antibiotics or anti-inflammatory drugs or changes in bowel habits in the month preceding surgery and/or during the last month of the study (month before evaluation), and no compliance with pre- or post-surgery dietary prescription.

All selected patients performed an obesity control program including a caloric-restricted diet, exercise, and behavioral modification, before and after surgery. All first samples (0 M) were collected 1 week before surgery. Thereafter, patients started a liquid diet. On post-operative day 1, every patient restarted a liquid diet that continued for a month, and after that, the same solid calorie-restricted diet of the pre-surgical period was gradually initiated. All surgeries were performed laparoscopically following current guidelines [23]. For SG, a 36 Fr bougie was used to calibrate gastric resection which was started 5 cm proximally to the pylorus. For GB, a standard 150-cm alimentary limb was performed. No antibiotic prophylaxis was used. All post-operative periods were uneventful with no need for re-intervention or revisional surgery.

### Clinical and Biochemical Evaluations

Clinical and biochemical parameters evaluated are described in Tables 1 and 2 and included: weight, BMI, waist circumference measured at umbilical level, percent excess weight loss (%EWL), percent total weight loss (%TWL), and systolic blood pressure (SBP).

**Table 1:** Basal patients’ clinical and metabolic characteristics

Patients’ features	**Total** (*N* = 24)		**SG** (*N* = 14)			**GB** (*N* = 10)			*p*
	Mean (SD)	Min/max	Mean (SD)	Min/Max		Mean (SD)		Min/max	
Age (years)	41 (16.75)	35.0/51.75	40.8 (10.7)		35.0/45.0	44.4 (9.6)	39.0/52.0		0.399*
	*N*	%	*N*		%	*N*	%		
Gender (F)	16	66.7	8		57.1	8	80.0		0.679**
Diabetes	6	25	3		21.4	3	30.0		1.000**
Hypertension	12	50	6		42.9	6	60.0		1.000**
Dyslipidemia	18	75	9		64.3	9	90.0		0.649**
OSA	4	16.7	4		28.6	0	0		0.098**

For age, mean, standard deviation (SD), minimum (Min), and maximum (Max) values are described; all other variables are presented as absolute and relative frequencies

*F* female, *Y* yes, *N* no, *SG* sleeve gastrectomy, *GB* gastric bypass, *N* sample size; *OSA* obstructive sleep apnea

*t-test

**Fisher’s exact test

**Table 2:** Metabolic impact of bariatric surgery for the global population sample

	**0 M**		**6 M**		*p*
	$\overline{x } (\text{sd})$	Min/max	$\overline{x } (\text{sd})$	Min/max	
Weight (kg)	124.0 (26.3)	93/224	94.2 (22.0)	70/179	< 0.001**
BMI (kg/m^2^)	44.0 (6.3)	36/67	33.4 (5.6)	27/53	< 0.001**
%EWL	–	–	58.0 (15.9)	26/89	–
%TWL		–	24.1 (5.7)	11/34	–
Waist (cm)	124.0 (14.3)	97/160	101.9 (13.2)	81/140	< 0.001*
SBP (mmHg)	147.0 (13.0)	125/175	128.0 (14.0)	102/159	< 0.001*
Total C (mg/dl)	201.0 (49.0)	122/298	199.0 (42.0)	142/299	0.788*
HDL C (mg/dl)	48.0 (15.0)	19/79	51.0 (13.0)	25/84	0.067*
LDL C (mg/dl)	140.0 (39.0)	76/212	142.0 (36.0)	95/238	0.794*
Triglycerides (mg/dl)	174.0 (221.0)	50/1181	100.0 (38.0)	56/219	0.002**
HbA1c (%)	5.9 (1.5)	5/12	5.4 (0.6)	5/8	< 0.001**
Glucose (mg/dl)	106.0 (37.0)	73/216	93.0 (20.0)	77/179	0.047**
Insulin (mIU/l)	34.4 (53.7)	6/270	10.7 (5.7)	4/27	< 0.001**
C peptide (ng/ml)	4.2 (3.5)	1/15	2.2 (1.0)	1/5	< 0.001**
Uric acid (mg/dl)	6.3 (1.3)	3.9/9.4	5.2 (1.3)	3.3/8.6	< 0.001*
hsCRP (mg/l)	1.1 (0.9)	0/4	0.4 (0.5)	0/2	< 0.001**
HOMA2-IR	3.1 (2.2)	1/10	1.6 (0.8)	1/4	< 0.001**

Results with statistically significant difference are highlighted in bold; *N* = 24.

*t-test.

**Wilcoxon.

Biochemical measurements, performed in blood samples drawn after a 10-h fasting period at 0 M and 6 M, included the following: fasting glucose; insulin and C-peptide; glycated hemoglobin (HbA1c); total, low-density lipoprotein (LDL), and high-density lipoprotein (HDL) cholesterol (C); triglycerides; uric acid; and high-sensitivity c-reactive protein (hsCRP). The homeostatic model assessment of insulin resistance (HOMA2-IR) was calculated using HOMA2 Calculator version 2.2.3 (http:/www.dtu.ox.ac.uk).

### Gut Bacterial Microbiome Analysis and Profiling

The gut bacterial microbiome structural diversity was determined by the 16S rRNA Next Generation Sequencing (NGS) approach. Stool samples were collected at home by patients a week before surgery (before starting the liquid diet, 0 M) and at 6 M using a stool collection tube with DNA stabilizer (Stratec®, Birkenfeld, Germany). Upon reception, samples were identified by code number and preserved at − 80 °C until processed. DNA extraction was performed with a PSP Spin stool DNA Plus kit (Stratec®, Birkenfeld, Germany). V4 hypervariable region of 16S rRNA was amplified using modified versions of the bacterial universal primers 515F e 806R [24]. Library preparation was performed according to the 16S Metagenomic sequencing Library Preparation (#150,442,223 Rev.B) Illumina protocol. Batches of 12 samples were sequenced in MiSeq equipment (Illumina) using a MiSeq Reagent Nano Kit V2, 500 Cycles (Illumina®, San Diego, CA, USA).

Raw data processing, clustering, and taxonomic annotation were performed with the Mothur package (version 1.47) (www.mothur.org) [25] and Silva reference file (release V138) [16]. All uninformative features, such as low-count and/or low-quality data, associated with sequencing errors and/or low-level contamination were removed with low count and low variance filters (Mothur package). Data normalization between samples was performed by data scaling using the total sum scaling [26].

Using clean, informative feature-normalized data sets, the online tool Microbiome Analyst [27], utilizing the R package DESeq2 [28], was used to perform differential abundance analysis. Fold changes for differentially abundant taxa were calculated, and statistical analysis was conducted to determine their significance. Statistical significance was set to a *p* value < 0.05 with an FDR < 0.1 (Benjamini and Hochberg false discovery rate).

To assess the impact of SG and GB on each sample microbiota diversity, alpha-diversity analysis was conducted. The relative abundances and frequencies of each operational taxonomic unit (OTU) of the prokaryotic community were measured with the Chao1 diversity index. Statistical significance was evaluated using a non-parametric Mann–Whitney test. To assess beta-diversity, which compares the similarity or dissimilarity between different samples, the relative abundance of different taxonomic ranks (such as genus and family) was used for non-metric dimensional scaling (NMDS) plots with Bray–Curtis dissimilarity. The statistical significance was assessed using permutational ANOVAs (PERMANOVAs).

All raw data sets were deposited in the NCBI database with the accession number PRJNA859272.

Reviewer link:

https://dataview.ncbi.nlm.nih.gov/object/PRJNA859272?reviewer=52adkral9h0np5jnblc391608q

### Expression of Immuno-inflammatory Response Genes

The expression of 17 genes involved in the inflammatory response was evaluated in patients’ peripheral blood mononuclear cells at 0 M and 6 M. Mononuclear cells were isolated from 3 ml of blood collected in ethylenediaminetetraacetic acid (EDTA) tubes by density gradient column performed with Ficoll-Paque™ Plus solution (GE Healthcare Bio-Sciences®, Uppsala, Sweden). Harvested cells were submitted to mRNA extraction using NZY Total RNA Isolation kit® (NZYTech®, Lisbon, Portugal), with a DNAase decontamination step, following the manufacturer’s instructions. Relative quantification of gene expression was performed with SALSA Multiplex Ligation-dependent Probe Amplification (MLPA) R009 Inflammation mRNA kit® (MRC Holland®, Amsterdam, Netherlands), following manufacturer instructions using a 3130 Genetic Analyzer® (Applied Biosystems®, USA) and GeneMapper software v.3.7. For relative quantification of mRNAs of the genes of interest, the peak area of each gene was divided by the peak area of the control gene, β_2_-microglobulin (*B2M*) [29].

### Statistical Analysis

The IBM® SPSS® v26 (Armonk, NY, USA) was used for the statistical analysis of clinical and biochemical parameters. Quantitative variables are described as mean ± standard deviation or median ± interquartile range according to the symmetry of the distributions. Categorical variables are described with frequencies and percentages. Comparison between SG and BG was performed using the *t*-Student and the Mann–Whitney tests according to the verification of the normality assumption. The normality assessment was conducted using the Shapiro–Wilk test. Matched variables were compared using the *t*-Student and the Wilcoxon tests. The association between categorical variables was evaluated with Fisher’s exact test. Spearman’s correlation was used to assess the correlation between quantitative variables, notably HOMA2-IR and hsCRP at 0 M and 6 M. The statistical significance level was set as 0.05.

## Results

Of the 42 initially selected patients, 18 were eliminated from the analysis by applying exclusion criteria. Of the remaining 24 patients included, 14 underwent SG and 10 GB. All patients were Caucasian. Table 1 describes patients’ clinical parameters and shows that at 0 M there were no statistically significant differences between SG and GB patients.

Table 2 describes all the evaluated outcomes related to surgery’s metabolic impact on the global population. A comparison of results at 0 M and 6 M showed a significant decrease in most markers associated with metabolic disruption except for all cholesterol-related parameters. Spearman’s correlation did not show a statistically significant association between hsCRP levels and HOMA2-IR (*p* = 0.428).

To compare the results of both surgeries, the difference in values obtained for each parameter at 0 M and 6 M was calculated for each patient (Table 3). Near significant decreases in insulin (*p* = 0.047) and uric acid (*p* = 0.047) were observed in GB, whereas SG provided a significant decrease in HOMA2-IR (*p* = 0.035). There were no statistically significant differences in the average values of %EWL or %TWL between surgeries. However, the number of patients reaching the major outcome of > 50%EWL was higher for GB: 9 patients (90%) vs 8 patients (57%), though not reaching statistical significance (*p* = 0.172). Also, all patients submitted to GB showed > 20%TWL compared to 10 patients (71.4%) in SG (*p* = 0.053), once more without statistical significance.

**Table 3:** Differences between 0 and 6 M parameters for each surgery

Parameters	**SG (*N* = 14)**		**GB (*N* = 10)**		*p*
	$\overline{x } (\text{sd})$	Min/max	$\overline{x } (\text{sd})$	Min/max	
Weight (kg)	30.9 (3.9)	25.0/37.0	28.8 (11.3)	10.0/46.0	0.550*
BMI (kg/m^2^)	11.2 (1.4)	9.2/13.3	10.0 (3.4)	3.9/14.7	0.266**
%EWL	63.8 (12.3)	41.1/85.5	53.1 (17.3)	26.2/89.1	0.102*
%TWL	26.0 (2.9)	21.9/32.7	22.4 (7.0)	10.0/46.0	0.111*
Waist (cm)	24.6 (5.7)	18.0/34.0	20.1 (11.8)	2.0/45.0	0.259*
SBP	20.1 (13.3)	0.0/38.0	18.2 (9.9)	3.0/31.0	0.686*
Total C (mg/dl)	5.9 (46.3)	− 65.0/98.0	− 1.0 (33.4)	− 99.0/37.0	0.676*
HDL C (mg/dl)	− 3.5 (6.2)	− 13.0/9.0	− 2.6 (9.1)	− 18.0/14.0	0.777*
LDL C (mg/dl)	4.8 (38.7)	− 57.0/77.0	− 7.5 (31.6)	− 79.0/34.0	0.398*
Triglycerides (mg/dl)	51.0 (65.7)	− 36.0/210.0	93.3 (264.5)	− 78.0/962.0	0.459**
HbA1c (%)	0.4 (0.6)	0.0/2.3	0.6 (1.5)	− 0.3/5.7	0.820**
Glucose (mg/dl)	12.7 (39.6)	− 34.0/124.0	13.8 (29.0)	− 20.0/85.0	0.776**
Insulin (mIU/l)	22.2 (24.5)	7.0/94.0	25.0 (70.8)	− 13.0/258.0	0.047**
C peptide (ng/ml)	2.6 (3.5)	− 0.9/12.6	1.6 (3.2)	− 0.5/11.9	0.063**
Uric acid (mg/dl)	0.8 (0.6)	0.1/2.0	1.4 (0.8)	0.2/2.6	0.047**
hsCRP (mg/l)	0.8 (0.7)	0.1/2.3	0.6 (1.2)	− 1.8/3.2	0.910**
HOMA2-IR	1.9 (2.3)	− 0.9/8.3	1.1 (1.8)	− 0.4/6.7	0.035**

Results with statistically significant differences are highlighted in bold. Negative values correspond to an increase of the plasma levels after the procedure (0–6 M).

*t-test.

**Mann–Whitney.

### Structural Diversity of the Gut Bacterial Microbiome

Regarding *Firmicutes* vs *Bacteroidetes* or vs *Proteobacteria* phylum ratios, we found a statistically significant decrease between 0 and 6 M specifically for the ratio *Firmicutes* vs *Proteobacteria* in patients that performed GB (average 15.8 and sd 12.3 at OM vs average 4.9 and sd 2.9 at 6 M; *p* < 0.05) (Table 1 supplementary material).

**Fig. 1:**
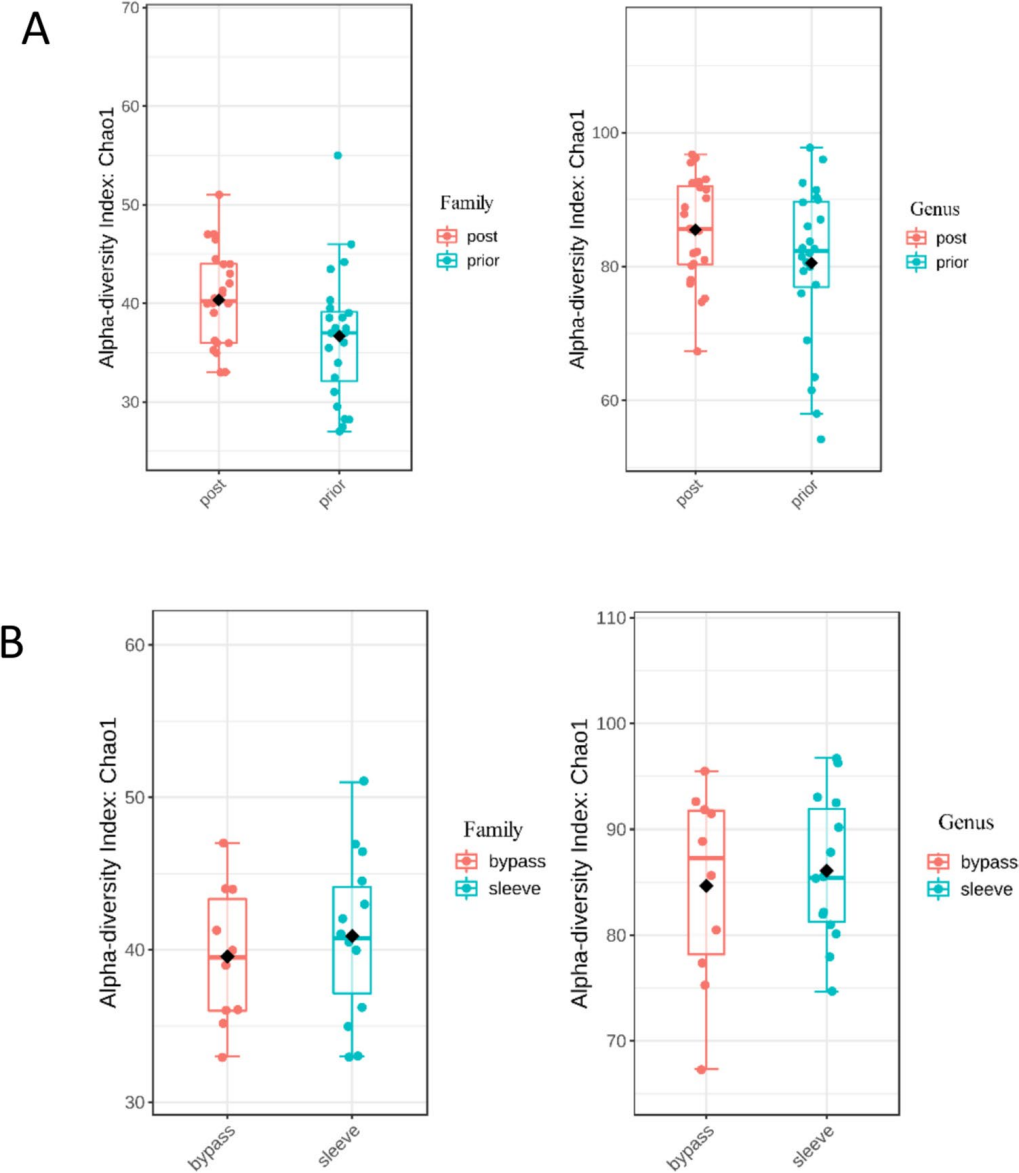
Alpha diversity (Chao1 index) of gut bacterial microbiome for the global population: **A** for prior (0 M) and post-surgery (6 M) samples for family and genus taxa (*p* = 0.026 and *p* = 0.184, respectively, and **B** by type of surgery, at 6 M, for family and genus taxa (*p* = 0.557 and *p* = 0.796, respectively). Statistical significance was evaluated using the Mann–Whitney test

Comparison of alpha diversity (measured by the Chao1 index) between samples before and after surgery for the whole population (Fig. 1A), showed a statistically significant increase for family-level taxa (*p* = 0.026), but not for the genus level (*p* = 0.184). When comparing after-surgery (6 M) samples between the two groups (SG and GB), there were no significant differences in alpha diversity either for family (*p* = 0.557) or genus (*p* = 0.796) taxa (Fig. 1B).

**Fig. 2:**
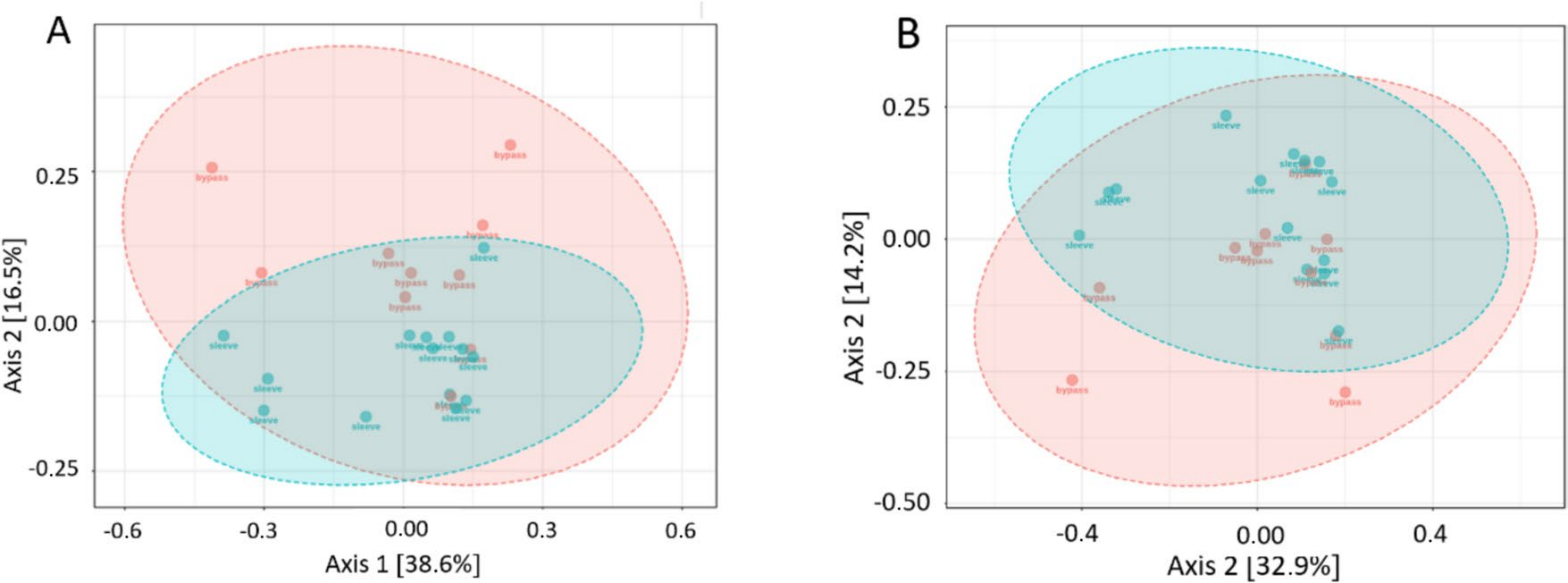
Beta diversity of gut bacterial microbiome assessed by non-metric multidimensional scaling (NMDS) plot with Bray–Curtis dissimilarity, showing near significant differences between SG and GB at 6 M (**A** family and **B** genus; *p* = 0.047 and *p* = 0.042 respectively). Orange spots correspond to GB samples and blue spots to SG samples

For beta diversity, no significant statistical differences were verified between pre- (0 M) and post-surgery (6 M) samples either for the global population or for each type of surgery. When comparing SG and GB samples, no difference in beta diversity was observed at 0 M. However, near significant differences were observed at 6 M at both the family (*p* = 0.047) and genus (*p* = 0.042) levels of taxonomy, as determined by PERMANOVA (Fig. 2).

**Fig. 3:**
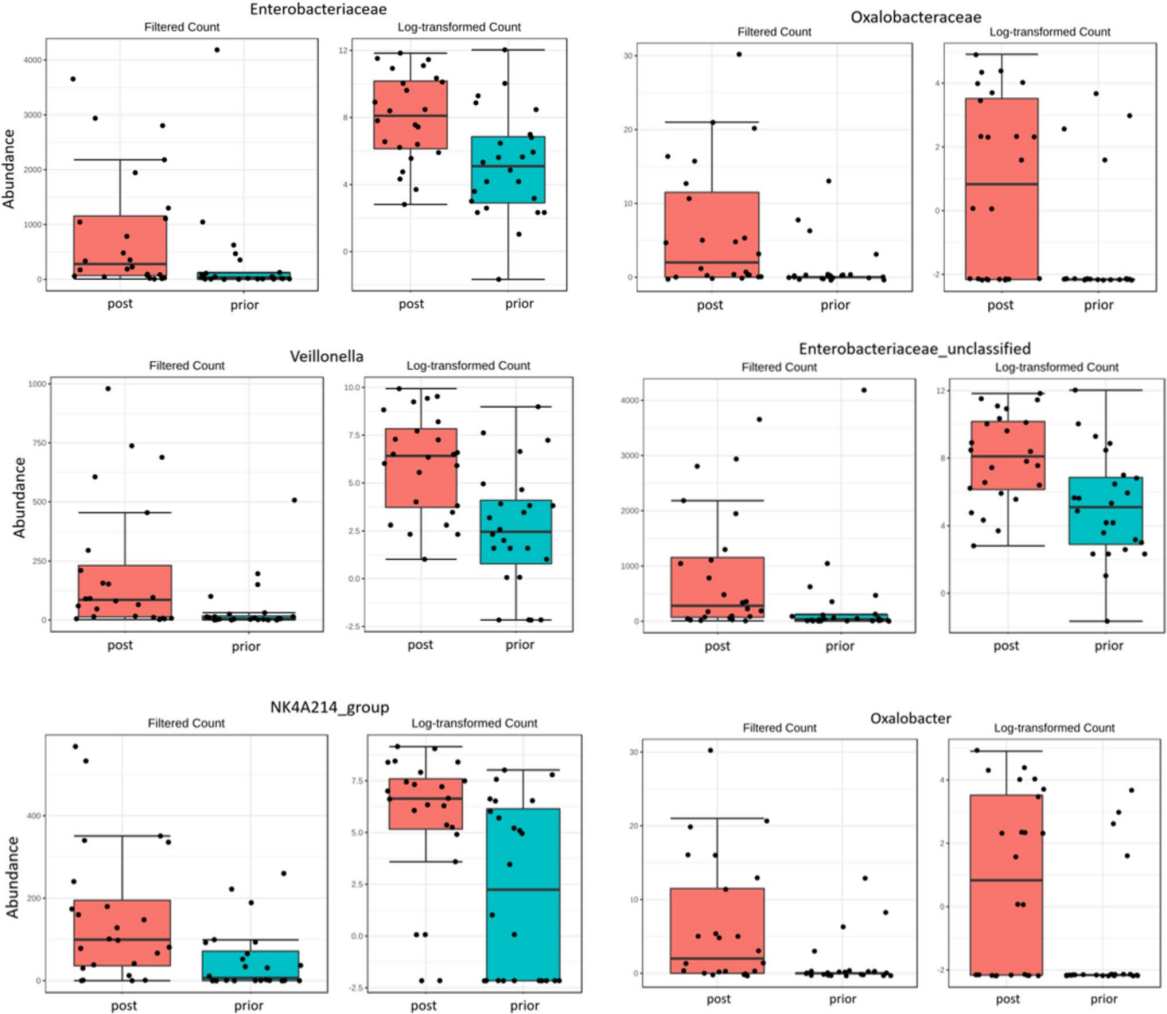
Differences in the relative abundance of families and genera level of gut bacterial microbiome at 0 M vs 6 M for the global population. Only statistically significant results are presented (Table 2 Supplementary material)

**Fig. 4:**
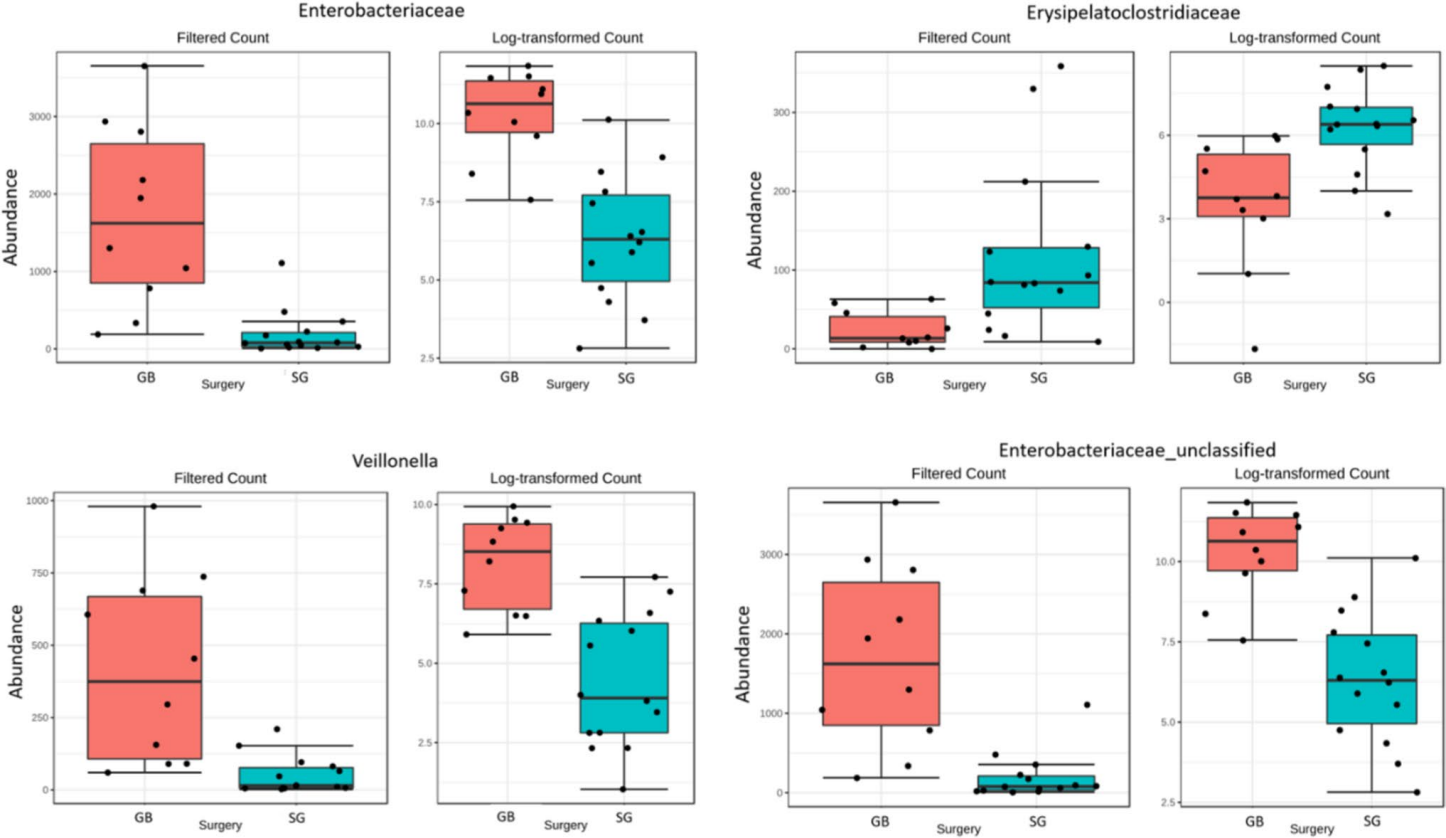
Differences in the relative abundance of families and genera level of gut bacterial microbiome between GB and SG at 6 M. Only statistically significant results are presented (Table 3 Supplementary material)

Analysis of relative abundance of individual taxonomic groups between 0 and 6 M, for the global population, showed significant differences in microbiota composition, with post-operative increased representation of *Enterobacteriaceae* and *Oxalobacteraceae* (both from phylum *Proteobacteria*) at family level and *Veillonella (*phylum *Firmicutes*), *Enterobacteriaceae_unclassified*, *Oxalobacter* and *NK4A214 group* (the late from phylum *Firmicutes*) at genus level (Fig. 3; Table 2 supplementary material). Comparison of relative abundances of gut microbiota at 6 M between SG and GB showed a higher representation of family *Enterobacteriaceae* and genera *Veillonella* and *Enterobacteriaceae_unclassified* in GB and higher representation of family *Erysipelatoclostridiaceae* (phylum *Firmicutes*) in SG (Fig. 4; Table 3 supplement material).

### Expression of Immuno-inflammatory Response Genes

Table 4 summarizes the variation of gene expression levels between 0 and 6 M for GB and SG patients. After sample processing, 23 complete sets of samples were analyzed, and one was excluded (low-quality sample). Unexpectedly, there was a global trend increase in values from 0 to 6 M for both interventions. Significant statistical differences were achieved by two marker genes (*NFkBIA* and *SCYA4*) for GB and by five marker genes (*IL4*, *NFkBIA*, *IL1RN*, *IL8*, *PDE4B*) for SG (Table 4).

**Table 4:** Comparison of the impact of GB and SG on immuno-inflammatory genes’ expression

Gene	GB		*p*	SG			*p*
	$\overline{x } (sd)$	Min/max		$\overline{x } (sd)$	Min/max		
*IL15* (0 M)	0.157 (0.078)	0.010/0.252	0.178^#^	0.146 (0.055)	0.074/0.250		0.735^#^
*IL15* (6 M)	0.200 (0.255)	0.040/0.916		0.203 (0.222)	0.032/0.682		
*IL15* (6–0 M)	0.040 (0.230)	− 0.100/0.660		0.060 (0.230)	− 0.080/0.560		*0.770*^§^
*IL4* (0 M)	0.134 (0.166)	0.048/0.600	0.646^#^	0.152 (0.132)	0.000/0.385		**0.018**^#^
*IL4* (6 M)	0.382 (0.411)	0.049/1.268		0.780 (0.560)	0.243/1.578		
*IL4* (6–0 M)	0.250 (0.320)	− 0.080/0.830		0.630 (0.520)	0.160/1.410		*0.064*^§^
NFkBIA (0 M)	1.501 (0.741)	0.665/2.516	**0.037**^#^	1.554 (1.251)	0.822/4.370		**0.018**^#^
NFkBIA (6 M)	2.027 (2.355)	0.052/8.498		3.774 (3.570)	1.058/9.208		
NFkBIA (6–0 M)	0.530 (2.070)	− 1.160/6.160		2.220 (2.840)	0.020/7.430		*0.051*^§^
*NFkB1* (0 M)	0.618 (0.211)	0.337/0.981	0.959^#^	0.611 (0.160)	0.372/0.798		0.128^#^
*NFkB1* (6 M)	0.975 (1.333)	0.061/4.691		0.941 (0.669)	0.178/2.337		
*NFkB1* (6–0 M)	0.360 (1.220)	− 0.280/3.810		0.330 (0.690)	− 0.190/1.850		*0.205*^§^
*TNF* (0 M)	0.116 (0.059)	0.047/0.233	0.799^#^	0.110 (0.038)	0.070/0.170		0.063^#^
*TNF* (6 M)	0.159 (0.148)	0.057/0.494		0.371 (0.271)	0.094/0.751		
*TN*F (6–0 M)	0.040 (0.150)	− 0.090/0.360		0.260 (0.300)	− 0.060/0.640		*0.079*^§^
*IL1B* (0 M)	0.055 (0.046)	0.000/0.135	0.799^#^	0.044 (0.035)	0.000/0.103		**0.043**^#^
*IL1*B (6 M)	0.067 (0.071)	0.020/0.255		0.283 (0.303)	0.023/0.793		
*IL1B* (6–0 M)	0.010 (0.060)	− 0.040/0.160		0.240 (0.290)	− 0.010/0.770		***0.040***^§^
*IL1RN* (0 M)	0.137 (0.049)	0.077/0.211	0.878^#^	0.177 (0.151)	0.061/0.511		**0.018**^#^
*IL1RN* (6 M)	0.151 (0.120)	0.033/0.456		0.547 (0.774)	0.108/2.272		
*IL1RN* (6–0 M)	0.010 (0.120)	− 0.140/0.300		0.370 (0.630)	0.000/1.760		***0.025***^§^
*IL8* (0 M)	0.133 (0.157)	0.008/0.525	0.333^#^	0.143 (0.203)	0.000/0.589		**0.028**^#^
*IL8* (6 M)	0.279 (0.397)	0.018/1.315		1.210 (1.022)	0.000/3.132		
*IL8* (6–0 M)	0.150 (0.440)	− 0.410/1.260		1.070 (0.890)	− 0.120/2.540		*0.051*^§^
*SCYA4* (0 M)	0.147 (0.077)	0.047/0.268	**0.017**^#^	0.160 (0.085)	0.044/0.267		**0.043**^#^
*SCYA4* (6 M)	0.208 (0.098)	0.084/0.347		0.269 (0.150)	0.070/0.439		
*SCYA4* (6–0 M)	0.060 (0.060)	− 0.050/0.140		0.110 (0.100)	− 0.040/0.260		*0.283*^§^
*SCYA3* (0 M)	0.058 (0.025)	0.018/0.100	0.074^#^	0.061 (0.024)	0.026/0.090		0.063^#^
*SCYA3* (6 M)	0.169 (0.265)	0.000/0.855		0.577 (0.489)	0.027/0.998		
*SCYA3* (6–0 M)	0.110 (0.250)	− 0.080/0.780		0.520 (0.490)	− 0.050/0.970		*0.172*^§^
*NFkB2* (0 M)	0.052 (0.032)	0.000/0.113	0.678^#^	0.053 (0.018)	0.027/0.076		0.866^#^
*NFkB2* (6 M)	0.048 (0.027)	0.000/0.093		0.069 (0.078)	0.000/0.228		
*NFkB2* (6–0 M)	0.000 (0.030)	− 0.040/0.040		0.020 (0.070)	− 0.070/0.150		*0.696*^§^
*SERPINB9* (0 M)	0.566 (0.173)	0.270/0.854	0.508^#^	0.556 (0.080)	0.498/0.723		0.612^#^
*SERPINB9* (6 M)	0.509 (0.162)	0.369/0.909		0.669 (0.392)	0.198/1.410		
*SERPINB9* (6–0 M)	− 0.060 (0.200)	− 0.440/0.200		0.110 (0.390)	− 0.300/0.830		*0.495*^§^
*THBS1* (0 M)	0.221 (0.145)	0.000/0.431	0.445^#^	0.323 (0.149)	0.180/0.623		0.237^#^
*THBS1* (6 M)	0.263 (0.214)	0.059/0.811		0.287 (0.189)	0.113/0.657		
*THBS1* (6–0 M)	0.040 (0.150)	− 0.170/0.380		− 0.040 (0.170)	− 0.210/0.320		*0.118*^§^
*LTA* (0 M)	0.033 (0.025)	0.000/0.057	0.515^#^	0.040 (0.006)	0.029/0.047		0.866^#^
*LTA* (6 M)	0.038 (0.019)	0.000/0.069		0.039 (0.039)	0.000/0.107		
*LTA* (6–0 M)	0.000 (0.020)	− 0.030/0.040		0.000 (0.040)	− 0.050/0.060		*0.558*^§^
*TNFRSF1A* (0 M)	0.178 (0.094)	0.000/0.357	0.066^#^	0.171 (0.075)	0.050/0.264		0.091^#^
*TNFRSF1A* (6 M)	0.111 (0.068)	0.000/0.243		0.101 (0.080)	0.000/0.214		
*TNFRSF1A* (6–0 M)	− 0.070 (0.110)	− 0.250/0.110		− 0.070 (0.090)	− 0.190/0.050		*1.000*^§^
*MIF* (0 M)	0.340 (0.117)	0.173/0.536	0.878^#^	0.333 (0.078)	0.266/0.485		1.000^#^
*MIF* (6 M)	0.348 (0.110)	0.213/0.481		0.351 (0.128)	0.192/0.569		
*MIF* (6–0 M)	0.010 (0.070)	− 0.100/0.120		0.020 (0.130)	− 0.130/0.210		*0.626*^§^
*PDE4B* (0 M)	0.360 (0.233)	0.099/0.808	0.139^#^	0.331 (0.149)	0.174/0.557		**0.028**^#^
*PDE4B* (6 M)	0.445 (0.182)	0.117/0.620		0.638 (0.353)	0.078/1.228		
*PDE4B* (6–0 M)	0.080 (0.210)	− 0.270/0.350		0.310 (0.240)		− 00.140/0.670	*0.064*^§^

^#^Wilcoxon test was used for comparing 0 M and 6 M values for each surgery

^§^Mann–Whitney test was used for comparing the mean variation (6–0 M) achieved by each patient between both surgeries (lower line *p* values in the right column). Expression levels are relative to *β*_2_-microglobulin gene expression level, used as a control gene for inter-sample homogenization. *N* = 23. Negative values correspond to a decrease in gene expression levels after the procedure (6–0 M). Transcript names are described in the supplementary material

To compare the impact of the two surgeries, the mean difference between 6 and 0 M values (6–0 M) of each patient was calculated, and the Mann–Whitney statistics was applied. There was a statistically significant difference between GB and SG patients in the levels of *IL1RN*, with a higher increase observed for SG patients (*p* = 0.025). The difference in levels of *IL1B* also achieved statistical significance (*p* = 0.04) (Table 4).

## Discussion

The impact of bariatric surgery in reducing weight and obesity-associated complications is well documented [30–32]. On the other hand, gut microbiota’s role in the stabilization and maintenance of the post-surgery new metabolic state is hampered by controversial results [15, 33, 34]. In this work, we analyzed the impact of two surgical approaches, GB and SG, in 24 patients with severe obesity, on metabolic parameters, gut bacterial microbiome, and variation in the blood cell expression levels of 17 genes related to immuno-inflammatory response.

Our results showed a favorable outcome 6 months after surgery, in line with current literature [30, 32]. The few cases in which the optimal result of > 50% EWL was not achieved were mainly in SG patients (6 patients with < 50% EWL), though with no statistically significant differences between surgeries. Serum biochemical markers’ improvement was adequate, showing a good metabolic outcome for both surgeries, except for cholesterol levels, for which total and LDL-cholesterol decreased and HDL-cholesterol increased, though differences were not statistically significant. An incomplete remission of dyslipidemia was reported by other authors after bariatric surgery [32, 34]. The difference in HOMA2-IR favoring SG over GB can be attributed to the small sample size and should be confirmed. Recent metanalysis shows that at long-term follow-up (more than 3 years), there is a slight, not clinically relevant, though statistically significant, higher weight loss for GB compared to SG, but apparently with no differences in quality of life and resolution of obesity complications [30–32].

Gut bacterial microbiome composition after surgery revealed an increase in alpha diversity for family-level taxa when analyzing the whole set of samples. Alpha diversity at 6 M was not influenced by the type of surgical intervention. However, for beta diversity, near-significant differences were observed between the post-operative SG and GB populations at both the family and genus levels of taxonomy. Other studies evaluating GB impact describe a trend to the restoration of bacterial richness and shifts in the representation of some phyla, with few differences between 6 and 12 months [14, 35]. For SG, the overall impact in bacterial GM seems to be similar, though less profound, and with differences in specific taxa fluctuations [15, 16, 36–38].

Analysis of the relative abundance of individual bacterial taxa, though revealing a high heterogeneity between samples of the same group, highlighted differences between the global population at 0 M and 6 M and between both surgeries at 6 M for specific taxa. For the global population, post-bariatric surgery GM showed increased representation of *Enterobacteriaceae* and *Oxalobacteraceae* (both from phylum *Proteobacteria*) at family level and *Veillonella (*phylum* Firmicutes*), *Enterobacteriaceae_unclassified*, *Oxalobacter*, and *NK4A214 group* (phylum *Firmicutes*) at genus level. Considering each surgery, relative abundances of gut microbiota at 6 M showed a higher representation of the family *Enterobacteriaceae* and genera *Veillonella* and *Enterobacteriaceae_unclassified* in GB and a higher representation of family *Erysipelatoclostridiaceae* (new family from phylum *Firmicutes* including members of the XVIII clostridial cluster) in SG.

*Veillonella* is an anaerobic gram-negative from the phylum *Firmicutes* that ferments lactate into acetate and propionate, which are short-chain fatty acids with relevant physiological roles, namely in maintaining colonic barrier integrity, local and systemic immunity, or regulation of appetite and energy balance [39, 40]. An increase in this bacteria population in GM has been linked to lower BMI in the general population and described in post-GB patients [14, 17]. In a study focusing on duodenal-jejunal liner placement, a reversible endoscopic procedure that mimics the effects of gastric bypass, the initial increase in *Veillonella* after placement of the liner was associated with better metabolic outcomes and the removal of the device resulted in a return to the previous microbiota and metabolic status [41]. Yet, there are also opposing results associating this genus with higher inflammatory status and insulin resistance in patients with obesity [42]. Cohort studies comparing bypass and sleeve have also shown an increase in *Veillonella* after GB but not after SG [15, 35, 38] in line with our results. *Enterobacteriaceae*, a large Gram-negative family from the phylum *Proteobacteria*, usually constitutes less than 1% of the healthy gut microbiota. *Enterobacteriaceae* encompasses a diversity of bacterial species including *Escherichia coli* (*E. coli*), which have relevant roles in shaping the gut anaerobic environment, producing vitamins, and protecting against pathogenic infections [43]. In literature, an increase in bacteria from the *Enterobacteriaceae* family is also more frequently reported for post-GB GM [14, 15]. Reduced colonization by *Oxalobacter formigenes*, a commensal Gram-negative anaerobic bacteria with oxalate metabolizing properties, has been associated with the increased risk of nephrolithiasis and hyperoxaluria described for GB, though the association remains controversial [44]. In our study, the increase in genus *Oxalobacter was* only statistically significant when considering the whole group of patients. Contrarily to other authors, we did not find significant differences in the relative abundances of butyrate-producing bacteria from phylum *Firmicutes* such as *Ruminococcus*, *Clostridium*, or *Coprococcus*, frequently described as being decreased after SG and GB [14, 15, 36]. We also did not find a greater abundance of *Akkermansia* described in some cohorts after GB [14, 15] or after SG [15, 38]. In our cohort, GB patients showed an inversion of the *Firmicutes/Proteobacteria* ratio, favoring *Proteobacteria,* not seen in the SG patients, results in line with other authors [4–8].

Differential gene expression analysis of gut mucosa before and after GB and subsequent gene set enrichment analysis suggest the involvement of pathways related to extracellular matrix organization, signal transduction, and metabolism [45]. To our knowledge, there are no reports on immuno-inflammatory mRNA gene expression in mononuclear blood cells of patients undergoing obesity surgery. This approach was meant to assess the impact on the immuno-inflammatory systemic response. Unexpectedly, our patients displayed an increase in mRNA gene expression levels after surgery. However, this feature is apparently contradicted by the significant reduction in post-operative hsCRP levels. Though there is not a direct linear correlation between gene expression and protein expression and function, due to post-transcriptional and post-translation processing, for some transcripts, the expression levels more than doubled. Even more interestingly, the significant increase in many of the mRNAs occurred mainly in SG patients. These facts suggest that bariatric surgery may have a positive impact on systemic immune response. In fact, though obesity-associated metabolic syndrome leads to a moderate increase in plasma biomarkers of inflammation, many animal and human studies also show that obesity impairs the effectiveness of the immune response to pathogens [46]. Modifications in lymphoid tissue architecture and in lymphocyte populations have been described and may in part be driven by leptin and adiponectin [46]. So, the observed higher levels of some mRNAs could be signaling immune system reactivation. On the other hand, the main stimuli to the liver synthesis of hsCRP are IL6 (not evaluated), TNF, with stable expression, and IL1B, for which the increase was scarce and could have been antagonized by the increased availability of IL1RN. So, the observed increased levels of specific mRNAs are compatible with decreased levels of hsCRP after surgery.

The gut microbiome is host-specific and varies with geographical location, race, ethnicity, and diet [47, 48]. Moreover, research on the impact of obesity surgery in GM reveals great variability of results even considering family and genus levels, justifying the importance of gathering data from different populations for future meta-analysis. This study’s strengths include the strict selection of patients, with control of confounding variants such as differences in pre- and post-surgical diets, being a longitudinal study with paired sample analysis for each patient, comparison between SG and GB, and the results suggesting systemic reactivation of immune system response at the gene transcription level. Our study’s main limitation is cohort size. A shot-gun metagenome approach would also favor a more comprehensive analysis of microbiota structure, with the inclusion of data from *Archaea* and *Eukaria* groups that compose the gut microbiota, as well as important functional data, particularly at the gene functional level.

## Conclusions

We conclude that obesity surgery results in a healthier patient, improving metabolic syndrome and obesity complications. SG and GB have similar clinical and metabolic outcomes. Differences in gut bacterial microbiome profile were observed in our cohort, mainly for some bacteria strains previously reported as being linked to a healthier metabolic state. GB and SG exhibited different profiles in some taxonomic groups. The patient’s inflammatory state due to metabolic syndrome improves after surgery, with the suggestion of reactivation of immune response as shown by the increased expression of immuno-inflammatory genes in plasma leukocytes, mainly after SG.

## Supplementary Information

Below is the link to the electronic supplementary material.

Supplementary file1 (DOCX 21 KB)

## Data Availability

Regarding microbiome analysis: All raw data sets were deposited at NCBI database with the accession number PRJNA859272. Reviewer link: https://dataview.ncbi.nlm.nih.gov/object/PRJNA859272?reviewer = 52adkral9h0np5jnblc391608q.
