# Supplementary material for: Sleeve Gastrectomy and Gastric Bypass Impact in Patient’s Metabolic, Gut Microbiome, and Immuno-inflammatory Profiles—A Comparative Study
Source: Obes Surg. 2025 Jan 28;35(3):733–45. doi: 10.1007/s11695-025-07708-9 (PMC11906558; doi:10.1007/s11695-025-07708-9)
Supplement: Supplementary file 1 — Supplementary file1 (DOCX 21 KB) [file 11695_2025_7708_MOESM1_ESM.docx]

**Supplementary material**

Genes included in gene expression analysis*: IL15* – Interleukin 15; *IL4* – Interleukin 4; NFKBIA - NFKB Inhibitor Alpha; *NFKB1* - Nuclear Factor Kappa B Subunit 1; *TNF* - Tumor Necrosis Factor; IL1B – Interleukin 1B; IL1 RN - Interleukin 1 receptor antagonist; IL 8 – Interleukin 8; *SCYA4* or *CCL4* - (C-C Motif Chemokine Ligand 4); *SCYA3* or *CCL3* - (C-C Motif Chemokine Ligand 3); NFKB2 - Nuclear Factor Kappa B Subunit 2; SERPINB9 - Serpin Family B Member 9; *THBS1*- Thrombospondin-1; *LTA* - Lymphotoxin Alpha; *TNFRSF1A*- Tumor Necrosis Factor Receptor Superfamily Member 1A; MIF - Macrophage Migration Inhibitory Factor; *PDE4B* - Phosphodiesterase 4B.

**Table 1**. Comparison of *Firmicutes/Bacteroidetes* and *Firmicutes/Proteobacteria* ratios between 0M and 6M for both surgeries

| **Ratio** | **0M** | | **6M** | | |  | |
| --- | --- | --- | --- | --- | --- | --- | --- |
|  | **Average** | **sd** | | **Average** | **sd** | | ***P v***alue |
| ***Firmicutes/Bacteroidetes*** | 1.219 | 0.518 | | 1.255 | 0.453 | | 0.807 |
| SG | 1.059 | 0.435 | | 1.059 | 0.435 | | 0.142 |
| GB | 1.444 | 0.542 | | 1.194 | 0.545 | | 0.601 |
| ***Firmicutes/Proteobacteria*** | 17.614 | 10.877 | | 14.415 | 17.640 | | 0.463 |
| SG | 18.926 | 9.488 | | 21.186 | 20.425 | | 0.720 |
| GB | 15.775 | 12.334 | | 4.935 | 2.947 | | **0.019*** |

SG- Sleeve gastrectomy; GB- Gastric Bypass; * statistically significant

**Table 2**. Relative abundance of bacteria between 0M and 6M for the global population

| \| **Taxa** \| **Relative abundance** (%) \| \|  \| \| --- \| --- \| --- \| --- \| \|  \| **0M** \| **6M** \| ***p*** value \| \| **Family** \|  \|  \|  \| \| *Enterobacteriaceae* \| 0.1214 \| 1.0008 \| 0.002 \| \| *Oxalobacteraceae* \| 0.0000 \| 0.0104 \| 0.003 \| \| **Genus** \|  \|  \|  \| \| *Veillonella* \| 0.0261 \| 0.3651 \| < 0.001 \| \| *Enterobacteriaceae_unclassified* \| 0.0913 \| 0.8138 \| 0.002 \| \| *Oxalobacter* \| 0.0000 \| 0.0184 \| 0.003 \| \| NK4A214_group \| 0.0329 \| 0.5069 \| 0.004 \| |
| --- | --- | --- | --- | --- | --- | --- | --- | --- | --- | --- | --- | --- | --- | --- | --- | --- | --- | --- | --- | --- | --- | --- | --- | --- | --- | --- | --- | --- | --- | --- | --- | --- | --- | --- | --- | --- | --- | --- | --- | --- |

Only statistically significant results are presented

**Table 3**. Relative abundance of bacteria between SG and GB patients’ at 6M

| **Taxa** | **Relative abundance** (%) | | |
| --- | --- | --- | --- |
|  | **SG** | **GB** | ***p*** value |
| **Family** |  |  |  |
| *Enterobacteriaceae* | 0.2704 | 7.9494 | <0.0001 |
| *Erysipelatoclostridiaceae* | 0.4617 | 0.0826 | <0.002 |
| **Genus** |  |  |  |
| *Enterobacteriaceae_unclassified* | 0.2405 | 7.4178 | <0.0001 |
| *Veillonella* | 0.0257 | 1.5679 | 0.0005 |

Only statistically significant results are presented
